# Tyro3 and Gas6 are associated with white matter and myelin integrity in multiple sclerosis

**DOI:** 10.1186/s12974-024-03315-0

**Published:** 2024-12-13

**Authors:** Igal Rosenstein, Lenka Novakova, Hlin Kvartsberg, Anna Nordin, Sofia Rasch, Elzbieta Rembeza, Sofia Sandgren, Clas Malmeström, Stefanie Fruhwürth, Markus Axelsson, Kaj Blennow, Henrik Zetterberg, Jan Lycke

**Affiliations:** 1 Department of Clinical Neuroscience, Institute of Neuroscience and Physiology at Sahlgrenska Academy, University of Gothenburg, Blå Stråket 7, 413 45 Gothenburg, Sweden; 2 Region Västra Götaland, Department of Neurology, Sahlgrenska University Hospital, Gothenburg, Sweden; 3 Clinical Neurochemistry Laboratory, Sahlgrenska University Hospital, Mölndal, Sweden; 4 Department of Psychiatry and Neurochemistry, Institute of Neuroscience and Physiology, University of Gothenburg, Mölndal, Sweden; 5 Paris Brain Institute, ICM, Pitié-Salpêtrière Hospital, Sorbonne University, Paris, France; 6 UK Dementia Research Institute at UCL, London, UK; 7 Department of Neurodegenerative Disease, UCL Queen Square Institute of Neurology, London, UK; 8 Hong Kong Centre for Neurodegenerative Diseases, Hong Kong, China; 9 Wisconsin Alzheimer’s Disease Research Center, University of Wisconsin School of Medicine and Public Health, University of Wisconsin-Madison, Madison, WI USA

**Keywords:** Innate immunity, Demyelination, Remyelination, Quantitative MRI, Biomarkers

## Abstract

**Background:**

The Gas6/TAM (Tyro3, Axl, and Mer) receptor system has been implicated in demyelination and delayed remyelination in experimental animal models, but data in humans are scarce. We aimed to investigate the role of Gas6/TAM in neurodegenerative processes in multiple sclerosis (MS).

**Methods:**

From a prospective 5-year follow-up study, soluble Gas6/TAM biomarkers were analyzed in cerebrospinal fluid (CSF) by enzyme-linked immunosorbent assay (ELISA) at baseline in patients with relapsing–remitting MS (RRMS) (n = 40), progressive MS (PMS) (n = 20), and healthy controls (HC) (n = 25). Brain volumes, including myelin content (MyC) and white matter (WM) were measured by synthetic magnetic resonance imaging at baseline, 12 months, and 60-month follow-up. Associations with brain volume changes were investigated in multivariable linear regression models. Gas6/TAM concentrations were also determined at 12 months follow-up in RRMS to assess treatment response.

**Results:**

Baseline concentrations of Tyro3, Axl, and Gas6 were significantly higher in PMS vs. RRMS and HC. Mer was higher in PMS vs. HC. Tyro3 and Gas6 were associated with reduced WM (β = 25.5, 95% confidence interval [CI] [6.11–44.96, p = 0.012; β = 11.4, 95% CI [0.42–22.4], p = 0.042, respectively) and MyC (β = 7.95, 95%CI [1.84–14.07], p = 0.012; β = 4.4, 95%CI [1.04–7.75], p = 0.012 respectively) at 60 months. Patients with evidence of remyelination at last follow-up had lower baseline soluble Tyro3 (p = 0.033) and Gas6 (p = 0.014). Except Mer, Gas6/TAM concentrations did not change with treatment in RRMS.

**Discussion:**

Our data indicate a potential role for the Gas6/TAM receptor system in neurodegenerative processes influencing demyelination and ineffective remyelination.

**Supplementary Information:**

The online version contains supplementary material available at 10.1186/s12974-024-03315-0.

## Introduction

Multiple sclerosis (MS) is an immune-mediated disease in which complex interactions between the adaptive and innate immune systems influence disease severity. By secreting pro-inflammatory cytokines, macrophages and microglia can contribute to the destruction of myelin and axons, as well as oligodendrocyte loss [1], which contributes to disability worsening in MS [2, 3]. Therefore, molecules that inhibit macrophage activation can influence the pathogenesis of MS.

Growth arrest-specific 6 (Gas6), a multi-modular protein, is a ligand that activates receptors belonging to the TAM receptor family (Tyro3, Axl, and Mer) [4–6]. Gas6/TAM interaction is involved in several physiological processes, including cell migration, adhesion, growth, and survival [7, 8]. Several experimental animal models have suggested an involvement of the Gas6/TAM system in autoimmune neuro-inflammatory conditions, including MS [9–13].

Mice lacking the TAM ligand Gas6 demonstrate heightened demyelination and delayed remyelination when exposed to cuprizone [14, 15]. Tyro3 is widely expressed in the central nervous system (CNS), particularly within white matter tracts [16], and is known to regulate myelination [13]. In the peripheral nervous system, Tyro3 regulates the thickness of myelin sheaths by influencing Schwann cells [17]. All three TAM receptors are expressed on oligodendrocyte progenitor cells (OPCs). However, while Axl and Mer are subsequently absent from the oligodendrocyte lineage, Tyro3 undergoes significant upregulation in newly formed and mature myelinating oligodendrocytes [14]. In experimental demyelination, there is a concurrent decrease in Tyro3 expression, mirroring the loss of myelin basic protein (MBP). However, Tyro3 expression rebounds with the onset of remyelination, suggesting a potential active role for Tyro3 in the myelin repair process [14, 18].

Hence, ample evidence from animal models suggests a role for the TAM receptor system in MS, but studies investigating concentrations of TAM receptors and Gas6 in people with MS are still lacking. Furthermore, associations of the TAM receptor system with clinically meaningful outcomes in MS are poorly investigated. Our aim was to study the association between TAM receptor/Gas6 ligand levels and degeneration and repair processes in a prospective cohort of patients with relapsing–remitting (RR) and progressive MS (PMS).

## Methods

### Study design

Patients with RRMS onset and patients with PMS, as well as healthy controls, were consecutively included in a cohort study at the Multiple Sclerosis Center of Sahlgrenska University Hospital, Gothenburg, Sweden between April 2014 and June 2016, previously described [19]. Patients with RRMS were prospectively followed for 60 months to evaluate biomarkers of neurodegeneration. Healthy controls were age-matched with the RRMS group. In the present study, inclusion criteria were diagnosis of RRMS according to the 2017 revised McDonald criteria [20], performed lumbar puncture with CSF sampling at baseline and at 12 months follow-up. Exclusion criteria were other concomitant neurological, ophthalmological, or inflammatory diseases. All PMS patients were inactive and did not have relapses within two years from inclusion. Data on CSF and MRI for the PMS group were available at baseline. RRMS and PMS patients were treatment-naïve at study inclusion and baseline sampling, and patients with RRMS started treatment with disease modifying therapy (DMT) after the baseline visit. The date of the onset of first MS symptoms, date of the diagnosis and comorbidities were recorded at the baseline visit. The following procedures were included at baseline, 12 months and 60 months follow-up: Expanded Disability Status Scale (EDSS), MS Functional Composite (MSFC), symbol digit modalities test (SDMT), paced auditory serial addition test (PASAT) and magnetic resonance imaging (MRI). RRMS patients were dichotomized based on evidence of disease activity (EDA)−3 status [21]. EDA-3 was defined as the occurrence of either clinical relapses, and/or confirmed disability worsening (CDW) that was sustained for at least 6 months, and new T1 gadolinium-enhanced lesions/new/newly enlarging T2W lesions during the study follow-up. A clinical relapse was defined as neurological signs and symptoms lasting at least 24 h and that could not be explained by another cause. [20] CDW was defined as increase in EDSS score by ≥ 1.5, ≥ 1 and ≥ 0.5 if baseline EDSS was 0, 1.0–5.0 and ≥ 5.5, respectively.

### Biochemical analysis

All biomarker analyses have been performed by certified laboratory technicians at the Clinical Neurochemistry Laboratory at Sahlgrenska University Hospital. The samples were handled according to the consensus protocol of the BioMS-EU network for CSF biomarker research in MS [22]. CSF samples were collected, processed onsite, aliquoted, and frozen at − 80 °C. All analyses were performed at room temperature. All CSF samples were analyzed blinded to treatment group in duplicate (Gas6, Tyro-3 and Mer) or triplicate (Axl) using commercially available ELISA kits (AXL # EHAXL ThermoFischer Scientific, Gas6 # BMS2291 ThermoFischer Scientific, TYRO-3 # BMS2287 ThermoFischer Scientific, MER # BMS2285 ThermoFischer Scientific) according to the manufacturer’s instructions with the exception of dilution factor which was evaluated separately for each analyte (final dilution in plate Axl 1:5, Gas6 1:30, Tyro-3 1:40, Mer 1:4). Longitudinal samples were analyzed on the same plate. The concentrations of CSF and serum GFAP and NfL, performed after defrosting, were measured with the Single Molecule Array (Simoa^®^) NEUROLOGY 2-PLEX B Kit, Product number: 103520, from Quanterix (Billerica, MA, USA) [23]. The LLoQ for serum GFAP and NfL was 29.4 pg/mL and 1.41 pg/mL, respectively. Serum samples of GFAP and NfL below the LLoQ level were designated the value of fLLoQ. The intra- and interassay coefficients of variation of all analyses were below 10%.

### Magnetic resonance imaging

Brain MRI was performed on a 3.0 Tesla MRI scanner (Philips Achieva dStream, head coil type with 16 coil channels) and conventional post-contrast T1-weighted, T2-weighted, fluid-attenuated inversion recovery and Synthetic (Sy) MRI sequences, were acquired. The SyMRI uses Quantification of Relaxation Times and Proton Density by Multiecho acquisition of a saturation-recovery using Turbo spin-Echo Readout (QRAPMASTER) approach described in detail previously [24] and is validated for clinical use for measuring brain parenchymal fraction (BPF) [25]. Recently, a rapid estimation of myelin for diagnostic imaging (*REMyDI*) [26] was validated and provides myelin content (MyC) of the brain. Further, we extracted gray matter (GM) and white matter (WM) volumes. The quantitative measures were created from R1, R2 and PD maps via SyMRI software (version 11.2; SyntheticMR, Linköping, Sweden). This segmentation is based on absolute values without any adjustment for lesion volume. BFP and MyC enable tracking global brain atrophy and global cerebral demyelination or remyelination in patients with MS. BPF and MyC have been demonstrated to have low interindividual variability (for BPF 0.2% [27] and for MyC 0.6% [26]).

### Statistics

Data are presented as mean ± SD or as median and interquartile range (IQR), as appropriate. The Mann–Whitney U test, unpaired T test, χ^2^ test, and Fisher’s exact test were used for group comparisons, as appropriate. The Spearman correlation coefficient was used to calculate the correlations between Gas6/TAM at baseline and follow-up as well as correlations with age. Correlations between Gas6/TAM and CSF and serum NfL and GFAP were also calculated with the Spearman correlation coefficient. The Kruskal–Wallis test with false discovery rate (FDR) for multiple comparisons (Two linear step up procedure of Benjamini, Krieger and Yekutieli) was used to compare baseline TAM receptor and Gas6 concentrations between the RRMS and PMS groups. To further investigate the interaction of Gas6/TAM receptors with age in the different groups, we performed analysis of covariance (ANCOVA), adjusting for age as a covariate.

Associations of TAM receptors and Gas6 ligand with brain volumes at baseline were investigated in multiple regression models adjusted for age, sex, and disease duration. Associations with brain volumes at 12-month follow-up as well as change in brain volumes were investigated in multiple regression models adjusted for age, sex, disease duration, and treatment category (low efficacy and high efficacy DMTs). We calculated WM, MyC, GM and BPF change (Δ) between baseline (month 0) and 60 months. Delta values were used as outcome endpoints in multivariable linear regression models as described above. All linear regression models were checked for normality of residuals by visual inspection of residual scatter and PP plots. We also calculated changes in MyC at 60 months minus baseline, and patients with positive values were classified as remyelination whereas negative values were classified as ineffective remyelination. We compared differences of Gas6/TAM concentrations between these two groups with the Mann–Whitney U test.

To investigate the influence of treatment on Gas6/TAM in RRMS, baseline concentrations were compared with levels at 12 months by paired T tests. To investigate treatment response, the RRMS cohort was further dichotomized into those who fulfilled no EDA-3 (NEDA-3) criteria at 12 months from those who did not. Concentrations at baseline and 12 months were compared in the NEDA-3 and EDA-3 groups by multiple paired T tests. DMTs were categorized into high-efficacy (natalizumab, fingolimod) and low-efficacy (interferon, teriflunomide, dimethyl fumarate). For sensitivity analyses, we compared Gas6/TAM concentrations at baseline and 12 month follow-up in both treatment groups, and compared concentrations between the low-efficacy and high-efficacy groups at 12 month follow-up. Multiple comparisons were corrected using the Holm-Sidák method and the adjusted p value was determined and reported.

Statistical significance was assumed at *p* < 0.05 unless otherwise specified. All statistical analyses and figures were performed/created with IBM SPSS version 28.0.1.0 (Armonk, NY: IBM Corp. 2011) and GraphPad prism version 10.2.0 unless otherwise specified.

### Ethical standards

All patients participated voluntarily in the study and provided written informed consent. The study conformed to the Code of Ethics of the World Medical Association (Declaration of Helsinki). The Regional Ethics Review Board in Gothenburg, Sweden, approved the study (Reference number 895-13).

## Results

### Demographic and clinical characteristics of the study cohort

The study population consisted of 40 patients with early RRMS (82.5% female) and 20 inactive PMS patients (60% female), as well as 25 HC (60% female) (Table 1). Patients with PMS were older than RRMS patients (median [IQR] age in years 49.5 [48–54.5] vs. 34 [25.25–42.75], p < 0.001), had longer disease duration (p < 0.001), and higher disability (p < 0.001). The age of HC did not differ significantly from RRMS (Demographic and clinical characteristics of the study population are presented in Table 1.

**Table 1:** Clinical and demographical characteristics of MS patients and healthy controls included in the study

	Relapsing–remitting MS (n = 40)	Progressive MS (n = 20)	Healthy controls (n = 25)
Age, y, median (IQR)	34 (25.25–42.75)	49.5 (48–54.5)	28 (25.5–32)
Sex (F), n (%)	33 (82.5)	12 (60)	15 (60)
Disease duration, y, median (IQR)	0 (0–1)	16 (5.75–24.25)	–
BL EDSS, median (IQR)	2 (1–2.9)	6 (4–6.5)	^**−**^
Last EDSS, median (IQR)	1.5 (0–2)	–	–
BL SDMT, median (IQR)	58 (51–68)	–	–
12 m SDMT, median (IQR)	63 (57.5–71)	–	**–**
BL PASAT, median (IQR)	47 (38.5–53)	–	**–**
12 m PASAT, median (IQR)	56 (51–59.25)	–	**–**
Relapse within 3 months from baseline, n (%)	25 (62.5)	–	–
Disease activity during follow-up, n(%)	9 (22.5)	–	–
DMT, n (%) No DMT Interferon Teriflunomide Dimethyl Fumarate Fingolimod Natalizumab	3 (7.5) 2 (5) 4 (10) 16 (40) 3 (7.5) 12 (30)	– – – – – – –	– – – – – – –

MS, multiple sclerosis; IQR, interquartile range; y, years; F, female; BL, baseline; EDSS, expanded disability status scale; SDMT, single digit modalities test; PASAT, paced auditory serial addition test; DMT, disease modifying therapy; m, month

Data are shown as median and interquartile range unless otherwise specified

### Tyro3 and Gas6, but not Axl and Mer, are higher in PMS and correlate with markers of neuronal injury

**Fig. 1:**
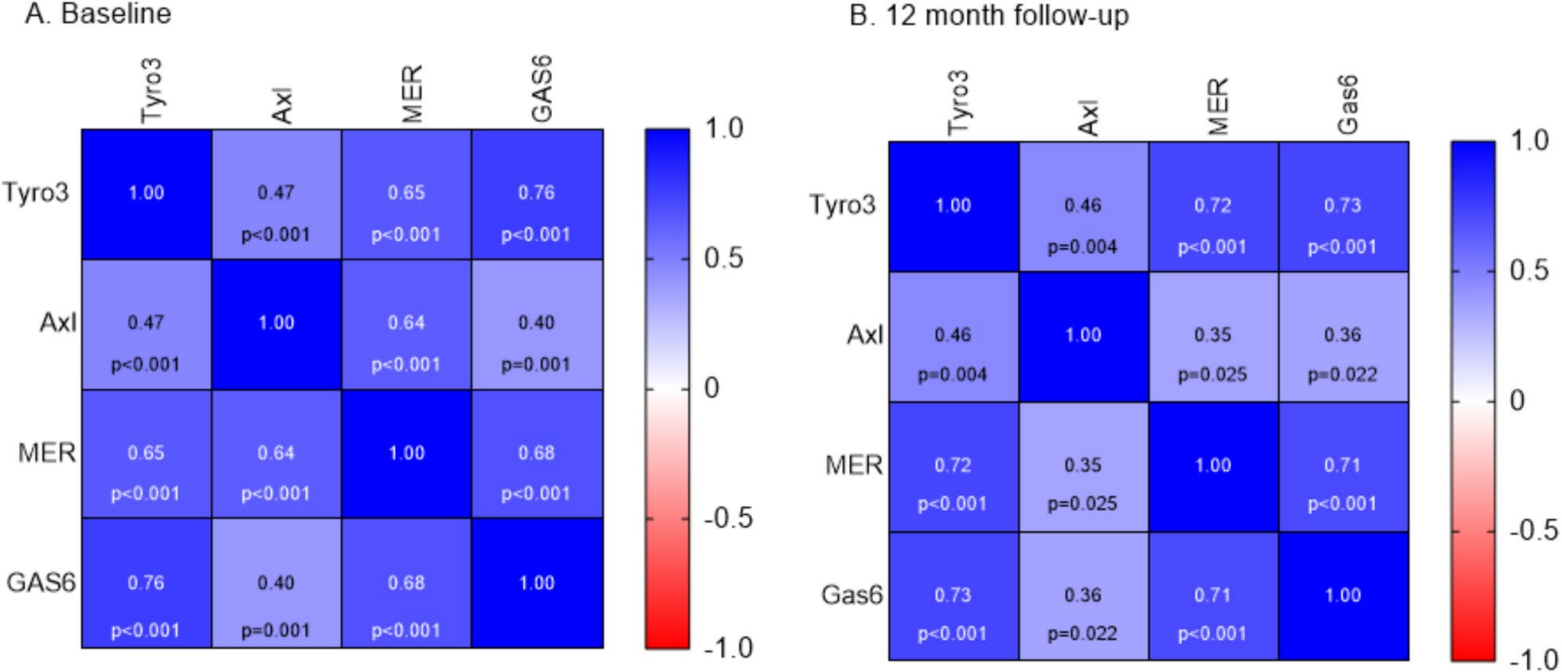
Heatmap correlation matrix showing Spearman correlation coefficients of Gas6/TAM receptors and p values. **A** At baseline (n = 60; RRMS [n = 40], PMS [n = 20]); **B** At 12 month follow-up (RRMS [n = 40]). RRMS, relapsing–remitting multiple sclerosis; PMS, progressive multiple sclerosis

**Fig. 2:**
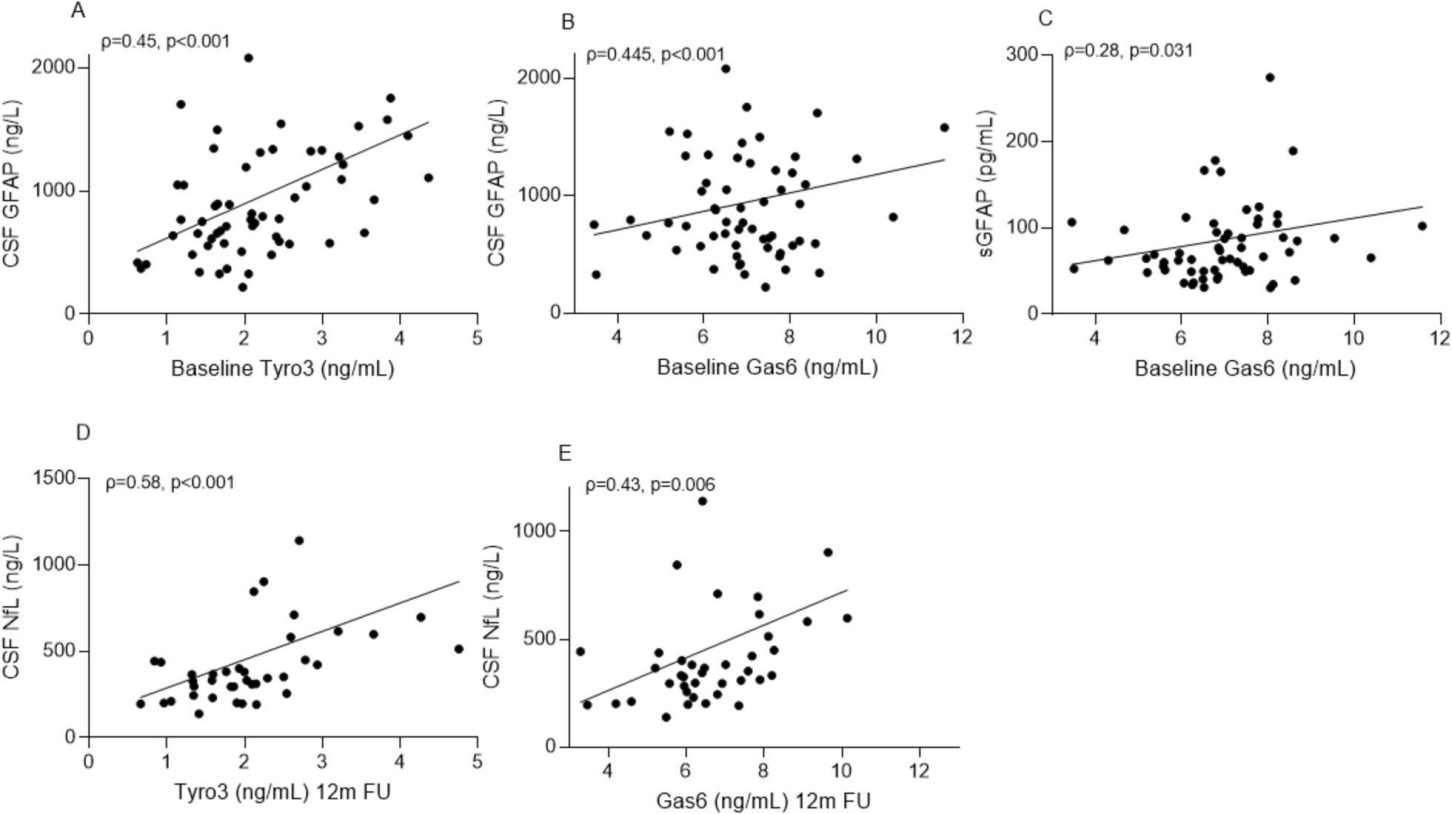
Associations of Tyro3 and Gas6 with biomarkers of neuroaxonal injury. **A** association of Tyro3 and CSF GFAP at baseline (n = 60); **B** association of Gas6 and CSF GFAP at baseline (n = 60); **C** association of Gas6 and sGFAP at baseline (n = 60); **D** association of Tyro3 and CSF NfL at 12 months follow-up (n = 40); **E** association of Gas6 and CSF NfL at 12 months follow-up (n = 40). CSF, cerebrospinal fluid; GFAP, glial fibrillary acidic protein; s, serum; NfL, neurofilament light

Baseline and follow-up biomarker data in RRMS and PMS are shown in Table 2. None of the Gas6/TAM biomarkers correlated with age nor were they associated with sex. Baseline (Fig. 1A) and follow-up (Fig. 1B) TAM receptors and the ligand Gas6 all correlated with each other, and the correlation between Tyro3 and Gas6 was the strongest (Baseline: r = 0.79, p < 0.001; Follow-up: r = 0.73, p < 0.001). Baseline concentrations of Tyro3 and Gas6 correlated with CSF GFAP (ρ = 0.45, p < 0.001; and ρ = 0.445, p < 0.001 respectively) (Fig. 2A, B). Moreover, Gas6 correlated with serum GFAP (sGFAP) (ρ = 0.28, p = 0.031) (Fig. 2C). In patients with RRMS, measurements at 12 months follow-up revealed a correlation between Tyro 3 and Gas6 and CSF NfL (ρ = 0.58, p < 0.001; and ρ = 0.43, p = 0.006 respectively) (Fig. 2D), and correlations of Tyro3 and Gas6 with CSF GFAP at 12 months follow-up remained significant (ρ = 0.41, p = 0.009; and ρ = 0.46, p = 0.003, respectively).

**Table 2:** Body fluid and imaging biomarkers of MS patients and healthy controls included in the study

	Relapsing–remitting MS (n = 40)	Progressive MS (n = 20)	Healthy controls (n = 25)
BL CSF Tyro3 ng/mL, median (IQR)	1.98 (1.61–2.44)	2.51 (1.5–3.24)	1.94 (1.26–2.64)
BL CSF Axl ng/mL, median (IQR)	1.79 (1.42–2.242)	2.22 (1.98–2.5)	1.71 (1.2–2.09)
BL CSF Mer ng/mL, median (IQR)	4.06 (3.29–5.14)	4.4 (3.85–5.67)	0.37 (0.31–0.44)
BL CSF Gas6 ng/mL, median (IQR)	6.76 (5.98–7.46)	7.58 (6.85–8.23)	6.34 (5.73–7.08)
12 m CSF Tyro3 ng/mL, median (IQR)	1.99 (1.41–2.59)	–	–
12 m CSF Axl ng/mL, median (IQR)	1.88 (1.27–2.08)	–	–
12 m CSF Mer ng/mL, median (IQR)	3.97 (3.12–4.87)	–	–
12 m CSF Gas6 ng/mL, median (IQR)	6.44 (5.87–7.67)	–	–
BL CSF NfL ng/L, median (IQR)	698 (513–1492)	633 (532–1057)	–
BL CSF GFAP ng/L, median (IQR)	649.3 (489.6–919.7)	1099.7 (817.5–1505.2)	–
BL sNfL pg/mL, median (IQR)	10.1 (7.5–16.9)	11.6 (10.3–14.5)	–
BL sGFAP pg/mL, median (IQR)	65.5 (52.3–84.8)	98.5 (67.2–120)	–
12 m CSF NfL ng/L, median (IQR)	348 (262.8–497)	–	**–**
12 m CSF GFAP ng/L, median (IQR)	671.3 (485.7–1056.5)	–	**–**
12 m sNfL ng/mL, median (IQR)	6.5 (5.01–9.05)	–	**–**
12 sGFAP ng/mL, median (IQR)	61.35 (48.5–88.25)	–	**–**
BL White matter volume (ml), median (IQR)	545.1 (522.6–608.4)	557.3 (487.5–605.4)	–
BL Gray matter volume (ml), median (IQR)	612.6 (573.6–650.3)	586 (528.8–657.8)	–
BL Myelin content (ml), median (IQR)	183.9 (173.7–204.6)	182.4 (156.8–209.3)	–
BL Brain parenchymal fraction (%)	0.89 (0.87–0.91)	0.82 (0.79–0.85)	–
60 m White matter volume (ml), median (IQR)	551.6 (509.9–607.9)	–	–
60 m Gray matter volume (ml), median (IQR)	581 (544.4–613.3)	–	–
60 m Myelin content (ml), median (IQR)	181.9 (166.9–208.2)	–	–
60 m Brain parenchymal fraction (%)	0.86 (0.84–0.89)	–	–

MS, multiple sclerosis; IQR, interquartile range; y, years; F, female; BL, baseline; CSF, cerebrospinal fluid; s, serum; NfL, neurofilament light; GFAP, glial fibrillary acidic protein; m, month

Data are shown as median and interquartile range unless otherwise specified

**Fig. 3:**
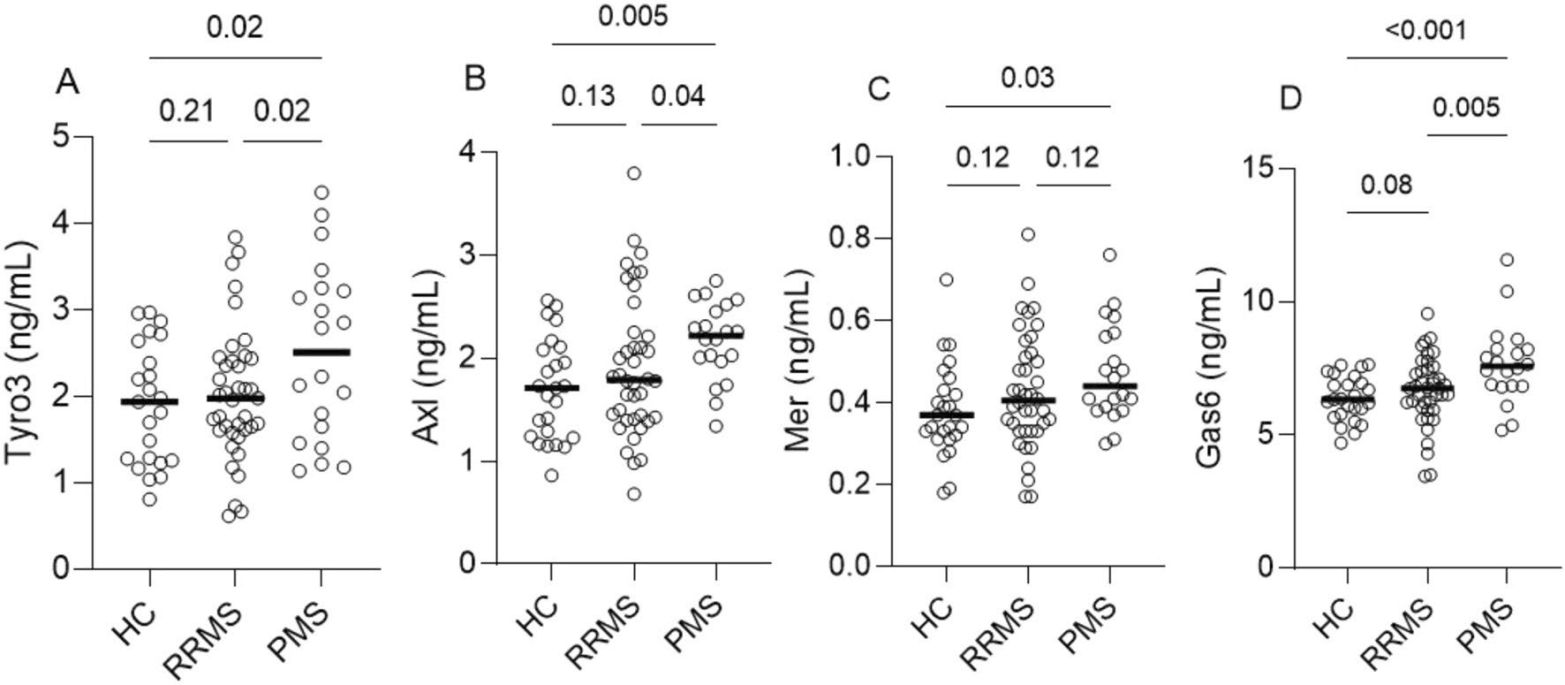
Scatter dot plots showing concentrations of Gas6/TAM biomarkers at baseline in patients with PMS (n = 20) compared to RRMS (n = 40) and healthy controls (n = 25). Line represents median and error bars represent interquartile range. Concentrations in the two groups were compared by unpaired T test. RRMS, relapsing–remitting multiple sclerosis; PMS, progressive multiple sclerosis

Baseline Tyro 3 concentrations were higher in PMS (median [IQR] 2.51 ng/mL [1.5–3.24]) compared to RRMS (1.97 [1.61–2.44]) (p = 0.02) and HC (1.94 [1.26–2.64]) (p = 0.02) (Table 2, Fig. 3A). Further, Axl was higher in PMS (2.22 [1.98–2.5]) vs. RRMS (1.79 [1.42–2.24]) (p = 0.04) and vs. HC (1.71 [1.23–2.09]) (p = 0.005) (Fig. 3B). Mer was higher in PMS (0.44 [0.38–0.56]) vs. HC (0.37 [0.31–0.44]) (p = 0.03) (Fig. 3C). Likewise, baseline levels of the soluble ligand Gas6 were higher in PMS (7.58 ng/mL [6.85–8.23]) compared to RRMS (6.76 [5.98–7.46]) (p = 0.005) and HC (6.34 [5.73–7.08]) (p < 0.001) (Fig. 3D). ANCOVA with age as a covariate largely confirmed these results. After adjustment for age, Axl, Mer, and Gas6 were higher in PMS vs HC (p = 0.042, p = 0.005, and p = 0.003, respectively). Tyro3 was numerically higher, but not significantly so (0.09). Mer was also higher in RRMS vs HC (p = 0.033) and Gas6 was higher in PMS vs RRMS (p = 0.007).

### Tyro3 and Gas6 are associated with myelin and white matter integrity

At baseline, Tyro3, Mer, and Gas6 positively associated with WM volume (Table 3). Gas6 was associated with MyC as well. However, at 12 months follow-up, none of the Gas6/TAM receptor biomarkers was associated with any brain volumes.

**Table 3:** Multiple linear regression models investigating the associations of Tyro3, Axl, Mer and Gas6 at baseline and 12 month follow-up with brain volumes and change in brain volumes in patients with RRMS

	**WM baseline**^**c**^		**MyC baseline**^**c**^		**GM baseline**^**c**^		**BPF baseline**^**c**^	
	β (95%CI)	p-value	β (95%CI)	p-value	β (95%CI)	p-value	β (95%CI)	p-value
Tyro3 (ng/mL)^a^	39.3 (9.65 to 68.9)	**0.011**	11.94 (− 0.05 to 23.9)	0.051	26.3 (− 3.7 to 56.3)	0.084	0.008 (− 0.007 to 0.022)	0.3
Axl (ng/mL)^a^	5.08 (− 32.1 to 42.3)	0.78	− 2.3 (− 16.4 to 11.8)	0.74	14.1 (− 17.2 to 45.4)	0.36	0.0003 (− 0.01 to 0.01)	0.96
Mer (ng/mL)^a^	185.8 (23.7 to 348)	**0.026**	55.5 (− 8.9 to 119.8)	0.089	43.5 (− 116.2 to 203.3)	0.6	0.045 (− 0.023 to 0.114)	0.18
Gas6 (ng/mL)^a^	19.7 (2.75 to 36.6)	**0.024**	6.74 (0.11 to 13.4)	**0.046**	2.9 (− 14.2 to 19.9)	0.73	0.006 (− 0.002 to 0.013)	0.13

	**WM 12 m follow-up**^**d**^		**MyC 12 m follow-up**^**d**^		**GM 12 m follow-up**^**d**^		**BPF 12 m follow-up**^**d**^	
Tyro3 (ng/mL)^b^	18.1 (− 23.3 to 48.4)	0.23	5.7 (− 6.5 to 17.9)	0.34	16.4 (− 14.5 to 47.3)	0.28	0.0003 (− 0.02 to 0.02)	0.97
Axl (ng/mL)^b^	15.8 (− 24.7 to 56.5)	0.3	6.9 (− 8.8 to 22.5)	0.37	− 0.23 (− 42.8 to 48.9)	0.99	0.00004 (− 0.02 to 0.02)	0.96
Mer (ng/mL)^b^	134.3 (− 58.03 to 326.6)	0.16	41.9 (− 35.9 to 119.7)	0.3	129.5 (− 51.7 to 310.7)	0.15	− 0.02 (− 0.11 to 0.073)	0.67
Gas6 (ng/mL)^b^	12.3 (− 3.7 to 28.2)	0.13	3.8 (− 2.7 to 10.3)	0.24	2.1 (− 14.4 to 18.6)	0.67	0.002 (− 0.006 to 0.01)	0.6

	**WMΔ**^**d**^		**MyCΔ**^**d**^		**GMΔ**^**d**^		**BPFΔ**^**d**^	
	β (95%CI)	p-value	β (95%CI)	p-value	β (95%CI)	p-value	β (95%CI)	p-value
Tyro3 (ng/mL)^a^	25.5 (6.11 to 44.96)	**0.012**	7.95 (1.84 to 14.07)	**0.012**	6.9 (− 9.24 to 23.04)	0.39	0.007 (− 0.001 to 0.026)	0.098
Axl (ng/mL)^a^	3.53 (− 18.54 to 25.61)	0.74	− 0.45 (− 7.43 to 6.53)	0.89	12.56 (− 3.4 to 28.53)	0.12	0.008 (− 0.005 to 0.21)	0.2
Mer (ng/mL)^a^	56.2 (− 51.9 to 164.24)	0.29	12.86 (− 21.6 to 47.3)	0.45	24.12 (− 58 to 106.25)	0.55	0.022 (− 0.25 to 0.68)	0.35
Gas6 (ng/mL)^a^	11.4 (0.42 to 22.4)	**0.042**	4.4 (1.04 to 7.75)	**0.012**	− 4.67 (− 12.86 to 3.53)	0.25	− 3223E−5 (− 0.005 to 0.005)	0.99

WM, white matter; MyC, myelin content; GM, gray matter; BPF, brain parenchymal fraction; CI, confidence interval

Bold text symbolizes p < 0.05

^a^Baseline

^b^12 month follow-up

^c^Multivariable models are adjusted for age, sex, and disease duration

^d^Multivariable models are adjusted for age, sex, disease duration, and DMT exposure

**Fig. 4:**
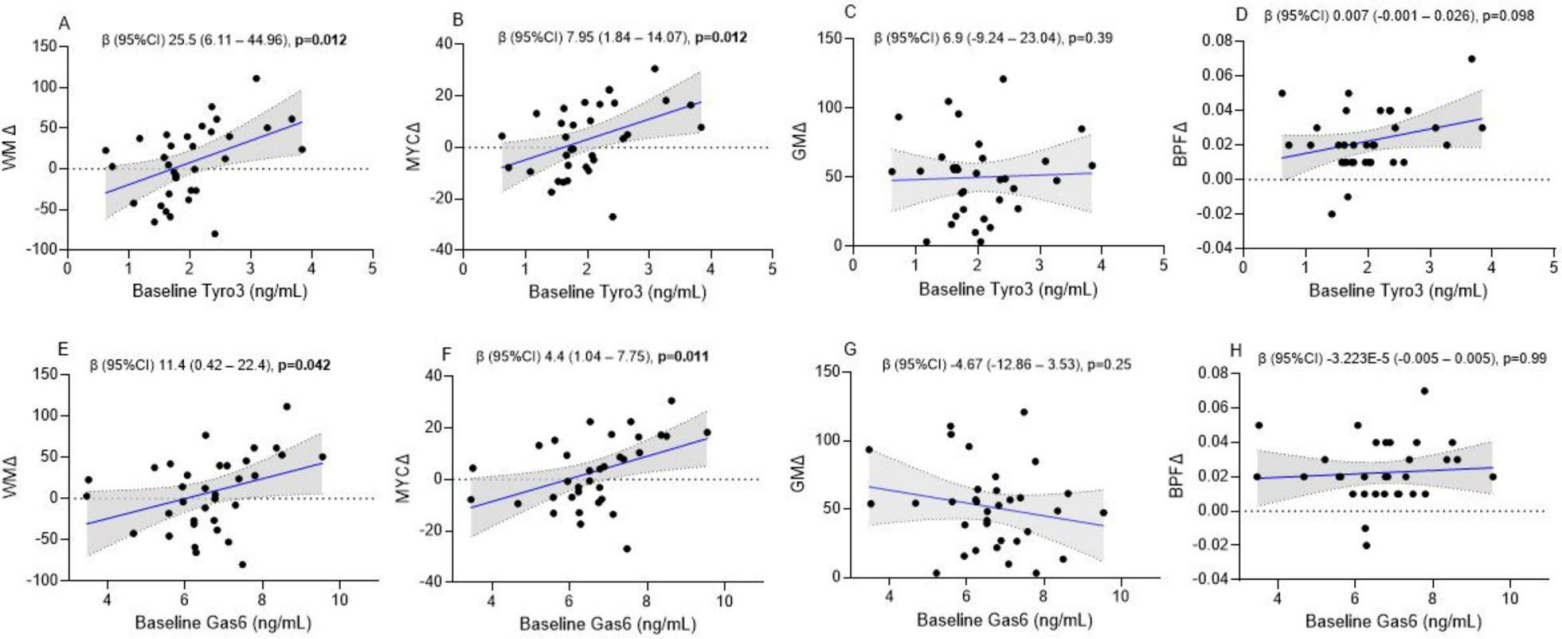
Associations of baseline Tyro3 and Gas6 with change in brain volumes in patients with RRMS (n = 40). Blue line represents mean and dotted black lines represent error bars. Results of multivariable linear regression models adjusted for age, sex, disease duration, and treatment category are shown in Table 2. RRMS, relapsing–remitting multiple sclerosis; WM, white matter; MYC, myelin content; GM, gray matter; BPF, brain parenchymal fraction

In a multiple linear regression models adjusted for age, sex, disease duration, and DMT exposure, baseline Tyro3 and Gas6 but not Axl nor Mer associated with WMΔ and MyCΔ (Table 3, Fig. 4). Neither Tyro3 nor Gas6 associated with GMΔ and BPFΔ (Fig. 4C, D, G–H). The complete results of all multiple linear regression models with all covariates are presented in supplementary Table 1.

RRMS patients who exhibited increased MyC values at 60 months (n = 18) compared to baseline had lower baseline Tyro 3 (median [IQR] 1.7 [1.53–2.02] vs. 2.3 [1.64–2.76], p = 0.033) and Gas6 (6.24 [5.68–6.77] vs. 7.2 [6.4–7.94], p = 0.01). There were no statistically significant differences for Axl (p = 0.96) nor Mer (p = 0.25).

### Associations of Gas6/TAM with clinical parameters

None of the Gas6/TAM biomarkers were associated with the EDSS at baseline and 12-month follow-up. Dichotomizing the RRMS cohort based on the cut-off EDSS ≥ 3, none of the Gas6/TAM biomarkers were shown to associate with Baseline EDSS and 12-month levels of Tyro3 were associated with greater reduction in PASAT but not SDMT score (ρ = 0.46, p = 0.019; and ρ = 0.41, p = 0.047 respectively). Gas6/TAM biomarker concentrations were not associated with either new/enlarging T2 lesions or presence of contrast-enhancing lesions on MRI.

### Tyro3 and Gas6 are not influenced by DMT and do not associate with treatment response

**Fig. 5:**
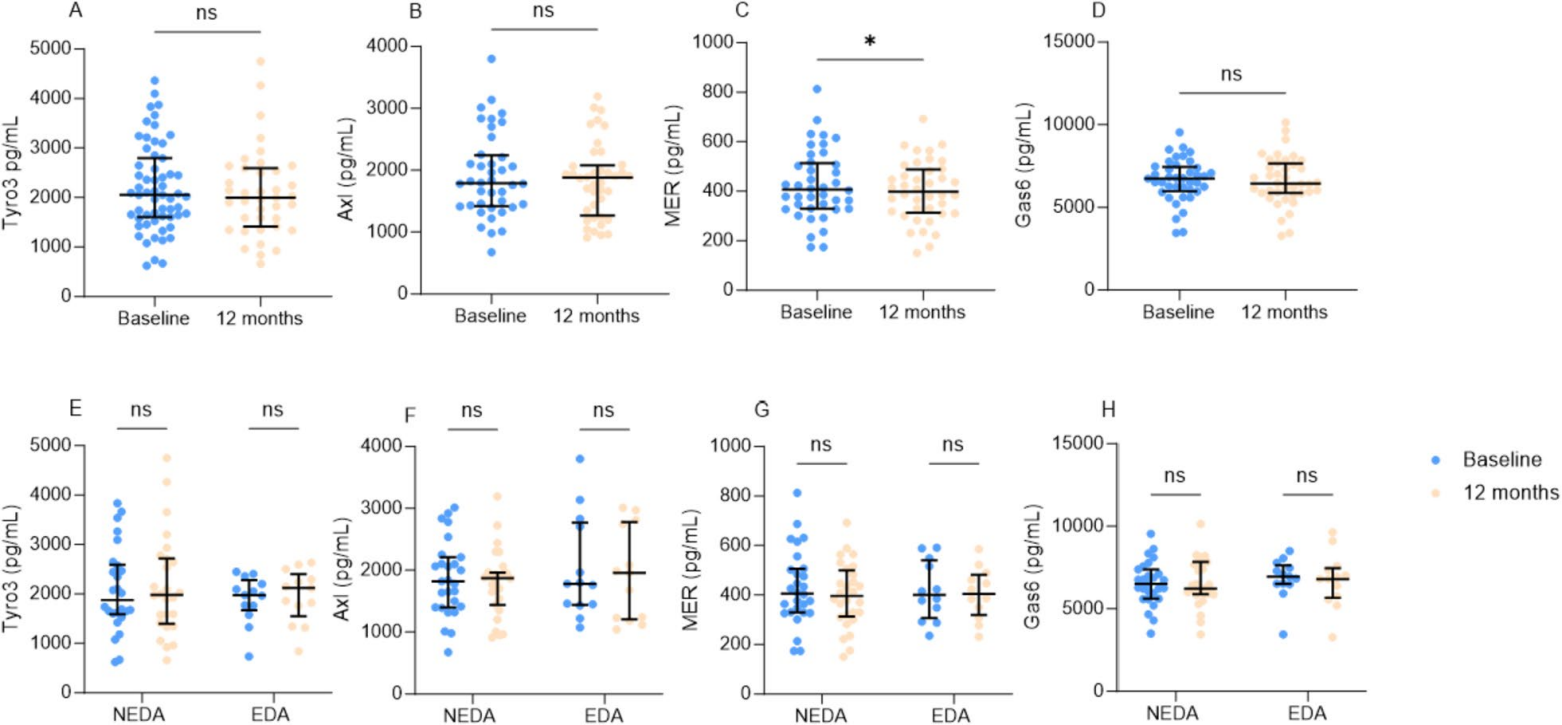
**A**–**D** Scatter dot plots showing comparisons of Gas6/TAM biomarker concentrations at baseline and 12 month follow-up. Comparisons were made by paired T tests. **E**–**H** Scatter dot plots showing comparisons of Gas6/TAM biomarker concentrations at baseline and 12 month follow-up dichotomized by EDA (n = 13) or NEDA (n = 27) status at follow-up. Comparisons were made by multiple T tests. Correction for multiple comparisons was made with the Hold-Sidak method and the adjusted p value was determined. EDA, evidence of disease activity; NEDA, no evidence of disease activity; ns, not significant. *p < 0.05

Mer concentrations were lower at 12 month follow-up compared to baseline (p = 0.04), but none of the other TAM receptors or Gas6 ligand significantly differed between baseline and follow-up (Fig. 5A–D). Thirteen RRMS patients had EDA-3 at 12-month follow-up, while 27 patients remained NEDA-3. Dichotomized by NEDA-3 status at follow-up, comparing Gas6/TAM concentrations at baseline and 12 months did not show any associations with treatment response (Fig. 5E–H). A sensitivity analysis performing the same tests in those who received only low-efficacy or only high-efficacy DMTs showed similar results (not shown). Gas6/TAM concentrations did not differ between the low-efficacy and high-efficacy groups at 12-month follow-up.

## Discussion

In the present study, we demonstrate a potential role of the Gas6/TAM receptor system in the loss of white matter integrity in MS patients, using CSF biomarker measurements of proteins related to these pathways. Essentially all Gas6/TAM biomarkers were higher in PMS compared to HC, and Tyro3, Axl, and Gas6 were higher in PMS vs. RRMS. In multiple linear regression models, baseline CSF concentrations of soluble Tyro3 and Gas6 were associated with greater reduction in WM and MyC at 60 months compared to baseline. In addition, RRMS patients who exhibited higher MyC at 60 months compared to baseline had lower baseline Tyro3 and Gas6, signifying an association with ineffective remyelination. Moreover, we found correlations between concentrations of Tyro3 and Gas6, and biomarkers of neuro-axonal damage (CSF NfL) and astrocyte activation (serum and CSF GFAP).

The association with GFAP was particularly consistent, further emphasizing findings from animal models [28] and fresh-frozen human MS brain tissue [29], in which the Gas6/TAM system was associated with axonal damage, inferior ability of remyelination and higher degree of neurodegeneration. GFAP is a marker of astrogliosis that has previously been proposed as a reliable biomarker for disease severity in MS [30, 31]. Further, sGFAP reflects astrocytic damage in patients with primary progressive MS [32], emphasizing its potential role in reflecting neurodegenerative processes in MS. Moreover, sGFAP has been demonstrated to associate with microstructural damage in normally-appearing white matter, as assessed by diffusion tensor imaging [33]. Recently, sGFAP has been shown to associate with progression independent of relapse activity in MS [34, 35]. Hence, the association of Tyro3 and Gas6 with CSF and serum GFAP may reinforce the notion of cross-talk between astrocytes, oligodendrocytes, and microglia in driving the chronic neurodegenerative processes (myelin damage and failure of repair) that characterize progressive MS.

Tyro3 has been previously implicated in the regulation of myelination in the CNS [13]. Knock-out mouse models have shown that loss of Tyro3 leads to delayed myelination and altered myelin thickness in the CNS [13]. Gas6 exhibits binding affinity and activation capabilities across all three TAM receptors; however, the heightened expression of Tyro3 on oligodendrocytes designates this receptor as the primary candidate for mediating the pro-myelinating effects induced by Gas6. It has been previously demonstrated that the absence of Tyro3 results in the abrogation of Gas6's pro-myelinating impact, leading to a delay in developmental myelination and the production of thinner-than-normal myelin. Notably, this effect is confined to the myelination process and is not attributable to alterations in the proliferation or differentiation of oligodendrocyte precursor cells. The loss of Gas6 has been demonstrated to be associated with delayed remyelination after a demyelinating injury induced by cuprizone [15].

We demonstrate an association of Tyro3, Gas6, and Mer with higher WM volume at baseline, and Gas6 was weakly associated with baseline MyC as well. However, this association was lost at 12 months follow-up, and baseline concentrations of Tyro3 and Gas6 were instead associated with greater reduction in WM volume and MyC at 60 months follow-up compared to baseline. Hypothetically, this may be explained neuroanatomically by greater concentrations of oligodendrocytes, astrocytes, and microglia in areas with larger WM volume and MyC, accounting for higher concentrations of soluble Gas6 and TAM receptors that are being released into the extracellular space. However, over time, this association is reversed, and higher baseline soluble Tyro3 and Gas6 are rather associated with greater reduction in WM and MyC, probably due to defective remyelination, partly because of TAM receptor inactivity.

Gas6-Tyro3 interactions have been demonstrated to be important in regulating remyelination and myelin sheath growth [13]. Recently, it was demonstrated that the promyelinating effects of Gas6 are dependent on the presence of Tyro3 [36]. It is thus not surprising that it is specifically soluble Tyro3 and the ligand Gas6 that particularly stand out in our analysis, and that both associate with loss of myelin and white matter volume as determined by quantitative MRI.

We found higher levels of Tyro3 and Gas6 in patients with progressive MS, implying that the Gas6/TAM receptor system may play a role in the degenerative processes driving progressive MS. Since Gas6/TAM did not correlate with NfL at baseline, it is likely that the Gas6/TAM system is not involved in the acute focal inflammatory neuroaxonal injury in MS, but rather involved in other neurodegenerative processes, related to ongoing demyelination and failure of remyelination. The lack of association between Gas6/TAM concentrations and new/enlarging T2 and contrast-enhancing lesions also reinforces this notion.

We found an association between baseline and 12 months Tyro3 concentrations and greater change (reduction) in PASAT score, indicating a possible role for Tyro3 in (re)myelination of fibres important for auditory information processing speed and ability. This preliminary finding encourages further explorations into the interaction of the Gas6/TAM receptor system and cognitive decline in MS.

However, our analysis did not reveal any convincing influence of DMT on Gas6/TAM concentrations at 12 months follow-up, although the levels of Mer were indeed reduced on a group level. Moreover, Gas6/TAM did not show a treatment response when dichotomizing the cohort according to NEDA-3 criteria or when comparing low- and high efficacy DMT treatment. However, we cannot rule out that the effect of treatment on Gas6/TAM is considerably slower and takes place over longer periods of time.

Most of the evidence on the role of the Gas6/TAM system is provided from experimental animal models of MS. To date, there has been limited replication of the aforementioned findings in individuals with MS. One study involving autopsy material from MS patients revealed an up-regulation of Axl and Mer in homogenates derived from chronic silent and chronic active lesions, respectively. [11] Additional insights into the association of Gas6/TAM with MS in humans have surfaced through genome-wide association studies (GWAS). Numerous single nucleotide polymorphisms (SNPs) situated within the *MerTK* gene have been identified as linked to susceptibility to MS [37, 38]. A distinctive SNP of the *MerTK* gene, denoted as rs7422195, was reported, which displays a discordant association with MS contingent upon the human leukocyte antigen (HLA)-DRB1*15:01 status [39]. Notably, rs7422195 demonstrates a protective effect in DR15 homozygosity, while exacerbating the disease in the absence of DR15. Furthermore, the minor allele of rs7422195 is correlated with an augmented gene and protein expression of MerTK in monocytes and CD4 + cells. More recently, this finding was confirmed [40], showing that DR15 and MerTK genotype independently influence proportions of CD14 + MERTK + monocytes in MS.

One study has previously investigated concentrations of Gas6/TAM in body fluids from MS patients [41], although this study assessed CSF and plasma concentrations of only Gas6. Findings from this study indicate that MS patients do not exhibit substantial alterations in plasma Gas6 concentration compared to controls. However, a dissociation between CSF and plasma was observed, wherein CSF Gas6 levels were higher in MS patients than in those with other non-inflammatory neurological diseases. Intriguingly, individuals experiencing more severe or prolonged relapses demonstrated lower CSF Gas6 concentrations, comparable to controls. In contrast, those with briefer and milder relapses displayed higher concentrations (almost twofold). Notably, CSF Gas6 concentration did not vary in relation to the completeness of recovery. Furthermore, neither plasma nor CSF Gas6 demonstrated associations with relapse rates or EDSS progression in a follow-up cohort. This is similar to a potentially protective role of increased serum TAMs in Alzheimer’s disease [42]. Patients with higher serum Gas6/TAM levels also demonstrate stronger immune regulation exerted by the TAM receptor system and promotion of phagocytosis and cell survival, resulting in preserved brain structure and delayed cognitive decline [43]. A very recently study published demonstrated a weak association between higher baseline CSF Gas6 and EDSS < 3 at diagnosis, but no association with follow-up EDSS, and a correlation between serum Gas6 with lower MS severity score [44]. These findings further emphasize the initial protective role of the Gas6/TAM system in the short term. Further, that study did not find differences in CSF concentrations of Gas6/TAM biomarkers when comparing patients on high-efficacy, low-efficacy, or no DMT, although patients on high-efficacy DMT exhibited somewhat lower serum levels of Axl. However, this analysis was cross-sectional and did not compare concentrations at baseline and follow-up (after DMT) in the same patients, as has been done in our study.

Our investigation has several limitations. This was a proof-of-concept study with a relatively small sample size, which limits statistical power and generalizability. Notwithstanding, we still could find meaningful differences in outcomes that should be validated in larger cohorts. The sampling before and after treatment initiation is only 12 months apart. However, the primary focus of the study was not the evaluation of treatment response nor the effect of DMT on biomarkers. Nonetheless, the follow-up time for MyC and WM was 60 months, which is sufficient to capture meaningful changes. Second, all our patients with RRMS are newly diagnosed, treatment naïve and have short disease duration at baseline. It may be valuable to investigate Gas6/TAM biomarkers in RRMS patients with longer disease duration in future studies to examine if there is increasing/decreasing trend in Gas6/TAM during disease course and to include treatments that could potentially have remyelinating effects. Further, the volumetric measures were performed after contrast, which is known to affect their values. However, the exact same protocol was used throughout the study and therefore this should not change the main results. Even though different dilution factors have been used for the different biomarkers, which might lead to quantitative proportional differences, this is not expected to influence the results of this study, as biomarker concentrations were not compared with each other.

In conclusion, we provide much needed evidence of CSF concentrations of Gas6, Tyro3, Axl, and Mer in people with MS and HC. Our findings underscore the potential role of Gas6/TAM receptor system, particularly Tyro3 and Gas6, in enhancing remyelination within the CNS in patients with early MS.

## Supplementary Information

Additional file 1

## Data Availability

No datasets were generated or analysed during the current study.
