# Supplementary material for: Tyro3 and Gas6 are associated with white matter and myelin integrity in multiple sclerosis
Source: J Neuroinflammation. 2024 Dec 13;21:320. doi: 10.1186/s12974-024-03315-0 (PMC11645787; doi:10.1186/s12974-024-03315-0)
Supplement: Supplementary file 1 — Additional file 1 [file 12974_2024_3315_MOESM1_ESM.docx]

| **Supplementary table 1.** Multiple linear regression models showing all covariates included in the models and investigating the associations of Tyro3, Axl, Mer and Gas6 at baseline and 12 month follow-up with brain volumes and change in brain volumes at last follow-up in patients with RRMS. | | | | | | | | |
| --- | --- | --- | --- | --- | --- | --- | --- | --- |
|  | **WM baseline*** | | **MyC baseline*** | | **GM baseline*** | | **BPF baseline*** | |
|  | β (95%CI) | p-value | β (95%CI) | p-value | β (95%CI) | p-value | β (95%CI) | p-value |
| **Tyro3 (ng/mL)^a^** | 39.3 (9.65 – 68.9) | **0.011** | 11.94 (-0.05 – 23.9) | 0.051 | 26.3 (-3.7 – 56.3) | 0.084 | 0.008 (-0.007 – 0.022) | 0.3 |
| Age (years) | -0.5 (-2.1 – 1.9) | 0.96 | -0.23 (-1.05 – 0.6) | 0.6 | -1.3 (-2.7 – 0.2) | 0.07 | -0.001 (-0.003 - -0.0004) | **0.008** |
| Sex (male) | -31.9 (-79.1 – 15.2) | 0.18 | -14.5 (-33.5 – 4.6) | 0.13 | -30.1 (-73.1 – 12.9) | 0.16 | 0.12 (-0.13 – 0.04) | 0.33 |
| Disease duration (years) | -2.7 (-4.9 – -0.4) | **0.023** | -0.7 (-1.7 – 0.2) | 0.12 | -0.36 (-2.9 – 2.3) | 0.7 | -0.002 (-0.004 - -0.001) | **0.003** |
| **Axl (ng/mL)^a^** | 5.08 (-32.1 – 42.3) | 0.78 | -2.3 (-16.4 – 11.8) | 0.74 | 14.1 (-17.2 – 45.4) | 0.36 | 0.0003 (-0.01 – 0.01) | 0.96 |
| Age (years) | 0.42 (-1.7 – 2.5) | 0.7 | -0.08 (-0.9 – 0.7) | 0.84 | -1.4 (-2.8 – 0.06) | 0.06 | -0.002 (-0.003 – -0.0004) | **0.005** |
| Sex (male) | -38.7 (-88.1 – 10.6) | 0.12 | -12.9 (-31.6 – 5.8) | 0.17 | -27.3 (-71.7 – 17.2) | 0.22 | 0.12 (-0.003 – 0.4) | 0.31 |
| Disease duration (years) | -1.8 (-4.1 – 0.55) | 0.13 | -0.67 (-1.6 – 0.24) | 0.14 | -0.5 (-3.2 – 2.3) | 0.72 | -0.002 (-0.004 - –0.001) | **0.003** |
| **Mer (ng/mL)^a^** | 185.8 (23.7 – 348) | **0.026** | 55.5 (-8.9 – 119.8) | 0.089 | 43.5 (-116.2 – 203..3) | 0.6 | 0.045 (-0.023 – 0.114) | 0.18 |
| Age (years) | 0.19 (-1.85 – 2.2) | 0.18 | -0.2 (-0.9 – 0.6) | 0.62 | -1.4 (-2.8 – 0.6) | 0.06 | -0.002 (-0.003 – -0.0004) | **0.005** |
| Sex (male) | -29.6 (-77.6 – 18.4) | 0.22 | -12.8 (-31.5 – 5.7) | 0.17 | -27.3 (-71.7 – 17.2) | 0.22 | 0.12 (-0.012 – 0.04) | 0.3 |
| Disease duration (years) | -2.06 (-4.3 – 0.16) | 0.068 | -0.6 (-1.5 – 0.3) | 0.17 | -0.47 (-3.2 – 2.25) | 0.73 | -0.002 (-0.004 - -0.001) | **0.003** |
| **Gas6 (ng/mL)^a^** | 19.7 (2.75 – 36.6) | **0.024** | 6.74 (0.11 – 13.4) | **0.046** | 2.9 (-14.2 – 19.9) | 0.73 | 0.006 (-0.002 – 0.013) | 0.13 |
| Age (years) | 0.2 (-1.86 – 2.24) | 0.85 | -0.2 (-0.9 – 0.6) | 0.61 | -1.4 (-2.8 – 0.06) | 0.06 | -0.002 (-0.003 – -0.0004) | **0.005** |
| Sex (male) | -31.5 (-79.6 – 16.6) | 0.2 | -13.4 (-32.1 – 5.1) | 0.15 | -27.3 (-71.7 – 17.2) | 0.22 | 0.012 (-0.012 -0.04) | 0.31 |
| Disease duration (years) | -2.5 (-4.8 - -0.18) | **0.035** | -0.76 (-1.5 – 0.14) | 0.095 | -0.46 (-3.1 – 2.25) | 0.73 | -0.002 (-0.004 – -0.001) | **0.003** |
|  | **WM 12m follow-up^§^** | | **MyC 12m follow-up^§^** | | **GM 12m follow-up^§^** | | **BPF 12m follow-up^§^** | |
| **Tyro3 (ng/mL)^b^** | 18.1 (-23.3 – 48.4) | 0.23 | 5.7 (-6.5 – 17.9) | 0.34 | 16.4 (-14.5 – 47.3) | 0.28 | 0.0003 (-0.02 – 0.02) | 0.97 |
| Age (years) | 0.63 (-1.9 – 3.2) | 0.61 | 0.014 (-1.05 – 1.07) | 0.97 | -2.2 (-4.005 – -0.32) | **0.023** | -0.001 (-0.002 – -0.0003) | **0.007** |
| Sex (male) | -12.2 (-73.3 – 48.8) | 0.7 | -6.8 (-31.1 – 17.4) | 0.56 | -116.8 (-168.3 – -65.3) | **<0.001** | 0.008 (-0.02 – 0.34) | 0.53 |
| Disease duration (years) | -3.3 (-9.1 – 2.4) | 0.24 | -1.2 (-3.5 – 1.16) | 0.31 | -0.4 (-4.9 – 5.7) | 0.8 | -0.0003 (-0.003 – 0.002) | 0.8 |
| DMT (he) | -29.1 (-76.7 – 18.5) | 0.22 | -13.5 (-32.6 – 5.76) | 0.16 | 2.4 (-44.03 – 48.7) | 0.92 | -0.013 (-0.035 – 0.008) | 0.2 |
| **Axl (ng/mL)^b^** | 15.8 (-24.7 – 56.5) | 0.3 | 6.9 (-8.8 – 22.5) | 0.37 | -0.23 (-42.8 – 48.9) | 0.99 | 0.00004 (-0.02 – 0.02) | 0.96 |
| Age (years) | 1.13 (-1.3 – 3.5) | 0.34 | 0.2 (-0.7 – 1.2) | 0.7 | -2.4 (-4.6 – -0.16) | **0.04** | -0.001 (-0.002 – -0.0003) | **0.016** |
| Sex (male) | -9.8 (-72.6 – 52.9) | 0.75 | -6.9 (-31.04 – 17.3) | 0.56 | -116.2 (-174.1 – -58.4) | **<0.001** | 0.009 (-0.02 – 0.04) | 0.52 |
| Disease duration (years) | -2.1 (-7.5 – 3.3) | 0.43 | -0.5 (-2.6 – 1.6) | 0.64 | 1.2 (-4.05 – 6.4) | 0.64 | -0.0003 (-0.003 – 0.002) | 0.78 |
| DMT (he) | -27.5 (-74.3 – 19.3) | 0.24 | -13.9 (-32.7 – 4.8) | 0.14 | 3.1 (-42.8 – 48.9) | 0.9 | -0.013 (-0.035 – 0.009) | 0.23 |
| **Mer (ng/mL)^b^** | 134.3 (-58.03 – 326.6) | 0.16 | 41.9 (-35.9 – 119.7) | 0.3 | 129.5 (-51.7 – 310.7) | 0.15 | -0.02 (-0.11 – 0.073) | 0.67 |
| Age (years) | 0.8 (-1.71 – 3.3) | 0.53 | 0.1 (-0.92 – 1.12) | 0.84 | -2.9 (-5.2 – -0,7) | **0.012** | -0.001 (-0.002 – -0.0001) | **0.03** |
| Sex (male) | -12.9 (-73.01 – 47.03) | 0.66 | -6.8 (-31.5 – 17.8) | 0.57 | -113.4 (-167.1 – -59.6) | **<0.001** | 0.008 (-0.2 – 0.035) | 0.54 |
| Disease duration (years) | -1.3 (-6.9 – 4.3) | 0.63 | -0.4 (-2.7 – 1.9) | 0.73 | 1.8 (-3.2 – 6,9) | 0.47 | -0.0004 (-0.003 – 0.002) | 0.73 |
| DMT (he) | -28.1 (-77.5 – 21.3) | 0.25 | -14.7 (-34.9 – 5.5) | 0.15 | -0.83 (-45.1 – 0.33) | 0.97 | -0.013 (-0.035 – 0.009) | 0.25 |
| **Gas6 (ng/mL)^b^** | 12.3 (-3.7 – 28.2) | 0.13 | 3.8 (-2.7 – 10.3) | 0.24 | 2.1 (-14.4 – 18.6) | 0.67 | 0.002 (-0.006 – 0.01) | 0.6 |
| Age (years) | 0.72 (-1.7 – 3.2) | 0.55 | 0.05 (-0.95 – 1.06) | 0.91 | -2.5 (-4.7 – -0.15) | **0.037** | -0.001 (-0.003 – -0.0001) | **0.013** |
| Sex (male) | -17.2 (-76.4 – 42.1) | 0.56 | -8.3 (-32.5 – 15.9) | 0.5 | -116.5 (-172.4 – -60.6) | **<0.001** | 0.008 (-0.02 – 0.035) | 0.53 |
| Disease duration (years) | -2.25 (-7.8 – 3.3) | 0.41 | -0.71 (-2.97 – 1.56) | 0.53 | 1.1 (-4.11 – 6.32) | 0.66 | -0.0003 (-0.003 – 0.002) | 0.74 |
| DMT (he) | -26.5 (-75.1 – 22.1) | 0.27 | -14.3 (-34.2 – 5.6) | 0.15 | 2.81 (-43.01 – 48,61) | 0.9 | -0.013 (-0.035 – 0.008) | 0.22 |
|  | **WMΔ^§^** | | **MyCΔ^§^** | | **GMΔ^§^** | | **BPFΔ^§^** | |
|  | β (95%CI) | p-value | β (95%CI) | p-value | β (95%CI) | p-value | β (95%CI) | p-value |
| **Tyro3 (ng/mL)^a^** | 25.5 (6.11 – 44.96) | **0.012** | 7.95  (1.84 – 14.07) | **0.012** | 6.9 (-9.24 – 23.04) | 0.39 | 0.007  (-0.001 – 0.026) | 0.098 |
| Age (years) | 0.85 (-0.65 – 2.4) | 0.25 | 0.31 (-0.17 – 0.8) | 0.19 | -0.78 (-1.96 – 0.39) | 0.19 | 0.0003 (-0.0004 – 0.001) | 0.4 |
| Sex (male) | -39.9 (-78.9 – -0.9) | **0.045** | -6.9 (-19.4 – 5.45) | 0.26 | -9.5 (-39.99 – 21.02) | 0.53 | -0.007 (-0.025 – 0.011) | 0.42 |
| Disease duration (years) | 0.75 (-2.81 – 4.32) | 0.66 | 0.18 (-0.95 – 1.31) | 0.74 | 0.16 (-24.12 – 21.67) | 0.91 | -0.00013 (-0.002 – 0.001) | 0.86 |
| DMT (he) | 25.4 (-3.9 – 54.7) | 0.086 | 6.4 (-2.91 – 15.71) | 0.15 | -1.22 (-24.12 – 21.68) | 0.91 | 0.006 (-0.005 – 0.016) | 0.28 |
| **Axl (ng/mL)^a^** | 3.53 (-18.54 – 25.61) | 0.74 | -0.45 (-7.43 – 6.53) | 0.89 | 12.56 (-3.4 – 28.53) | 0.12 | 0.008 (-0.005 – 0.21) | 0.2 |
| Age (years) | 1.24 (-0.23 – 2.72) | 0.095 | 0.44 (-43.4 – 12.32) | 0.26 | -0.83 (-1.95 – 0.3) | 0.057 | 0.0004 (-0.0003 – 0.001) | 0.26 |
| Sex (male) | -42.9 (-82.8 – -2.9) | **0.036** | -8.1 (-20.7 – 4.5) | 0.19 | -6.67 (-37.15 – 23.82) | 0.66 | -0.007 (-0.025 – 0.01) | 0.39 |
| Disease duration (years) | 1.74 (-1.75 – 5.24) | 0.31 | 0.44 (-0.66 – 1.55) | 0.42 | 0.6 (-2.07 – 3.3) | 0.65 | 0.0002 (-0.001 – 0.002) | 0.78 |
| DMT (he) | 27.25 (-2.54 – 57.04 | 0.071 | 7.3 (-2.12 – 16.71) | 0.12 | -5.6 (-28.4 – 17.16) | 0.62 | 0.008 (-0.006 – 0.014) | 0.39 |
| **Mer (ng/mL)^a^** | 56.2 (-51.9 – 164.24) | 0.29 | 12.86 (-21.6 – 47.3) | 0.45 | 24.12 (-58 – 106.25) | 0.55 | 0.022 (-0.25 – 0.68) | 0.35 |
| Age (years) | 1.06 (-0.42 – 2.55) | 0.15 | 0.4 (-0.09 – 0.85) | 0.11 | -0.77 (-1.98 – 0.44) | 0.2 | 0.0003 (-0.0003 – 0.001) | 0.33 |
| Sex (male) | -43.4 (-82.5 – -4.3) | **0.031** | -8.06 (-20.5 – 4.4) | 0.19 | -8.44 (-40.3 – 23.4) | 0.59 | -0.008 (-0.025 – 0.01) | 0.36 |
| Disease duration (years) | 1.94 (-1.51 – 5.4) | 0.26 | 0.51 (-0.59 – 1.61) | 0.35 | 0.46 (-2.3 – 3.3) | 0.74 | 0.0002 (-0.001 – 0.002) | 0.76 |
| DMT (he) | 25.6 (-3.83 – 55.01) | 0.086 | 6.76 (-2.61 – 16.14) | 0.15 | -4.9 (-28.8 – 19.02) | 0.67 | 0.008 (-0.005 – 0.021) | 0.24 |
| **Gas6 (ng/mL)^a^** | 11.4 (0.42 – 22.4) | **0.042** | 4.4 (1.04 – 7.75) | **0.012** | -4.67 (-12.86 – 3.53) | 0.25 | -3,223E-5  (-0.005 – 0.005) | 0.99 |
| Age (years) | 1.03 (-0.42 – 2.5) | 0.16 | 0.33 (-0.12 – 0.77) | 0.15 | -0.53 (-1.72 – 0.67) | 0.37 | 0.0004 (-0.0002 – 0.001) | 0.2 |
| Sex (male) | -42.23 (-80.93 – -3.5) | **0.034** | -7.6 (-19.5 – 4.3) | 0.2 | -8.95 (-40.67 – 22.76) | 0.56 | -0.008 (-0.026 – 0.01) | 0.37 |
| Disease duration (years) | 1.41 (-1.98 – 4.82) | 0.4 | 0.34 (-0.7 – 1.4) | 0.51 | 0.45 (-2.33 – 3.24) | 0.74 | 0.0001 (-0.001 – 0.002) | 0.85 |
| DMT (he) | 25.4 (-3.6 – 54.46) | 0.083 | 6.3 (-2.63 – 15.23) | 0.16 | -2.73 (-26.52 – 21.0.6) | 0.82 | 0.009 (-0.005 – 0.022) | 0.19 |
| Abbreviations: WM – white matter; MYC – myelin content; GM – gray matter; BPF – brain parenchymal fraction; CI – confidence interval; DMT – disease modifying therapy; he – high-efficacy.  ^a^ Baseline  ^b^ 12 month follow-up  ***** Multivariable models are adjusted for age, sex, and disease duration.  **^§^** Multivariable models are adjusted for age, sex, disease duration, and DMT exposure.  Bold text symbolizes p<0.05 | | | | | | | | |
